# BIM mediates synergistic killing of B-cell acute lymphoblastic leukemia cells by BCL-2 and MEK inhibitors

**DOI:** 10.1038/cddis.2016.70

**Published:** 2016-04-07

**Authors:** K Korfi, M Smith, J Swan, T C P Somervaille, N Dhomen, R Marais

**Affiliations:** 1 Molecular Oncology Group, Cancer Research UK Manchester Institute, University of Manchester, Manchester, UK; 2 Core Research Facilities, Cancer Research UK Manchester Institute, University of Manchester, Manchester, UK; 3 Leukemia Biology Group, Cancer Research UK Manchester Institute, University of Manchester, Manchester, UK

## Abstract

B-cell acute lymphoblastic leukemia (B-ALL) is an aggressive hematological disease that kills ~50% of adult patients. With the exception of some BCR-ABL1^+^ patients who benefit from tyrosine kinase inhibitors, there are no effective targeted therapies for adult B-ALL patients and chemotherapy remains first-line therapy despite adverse side effects and poor efficacy. We show that, although the MEK/ERK pathway is activated in B-ALL cells driven by different oncogenes, MEK inhibition does not suppress B-ALL cell growth. However, MEK inhibition synergized with BCL-2/BCL-XL family inhibitors to suppress proliferation and induce apoptosis in B-ALL cells. We show that this synergism is mediated by the pro-apoptotic factor BIM, which is dephosphorylated as a result of MEK inhibition, allowing it to bind to and neutralize MCL-1, thereby enhancing BCL-2/BCL-XL inhibitor-induced cell death. This cooperative effect is observed in B-ALL cells driven by a range of genetic abnormalities and therefore has significant therapeutic potential.

Acute lymphoblastic leukemia (ALL) is the most common childhood cancer and the third most common adult leukemia. Childhood ALL has good outcomes with 5-year survival rates of ~90%, whereas prognosis in older patients (15–65 years; ~40% of cases) is worse, with ~50% of patients dying from their disease. B-cell ALL (B-ALL) is the most common ALL (~70% of cases), so this disease has a clear unmet clinical need.^1, 2^ In addition to age, B-ALL outcome and response to therapy is determined by the genetic alterations that drive disease, with the *BCR-ABL1* and *MLL* rearrangement being associated with particularly poor prognosis.^3^ Chemotherapy remains first-line treatment in childhood and adult B-ALL^1^ and is combined with tyrosine kinase inhibitors (TKIs) in BCR-ABL1^+^ cases,^4^ but despite increased survival from intensive chemotherapy regimens, short- and long-term adverse effects are major drawbacks and the presence of chemoresistant subclones limits responses.^5^ Thus there is an urgent need for novel targeted therapies with improved efficacy and reduced toxicity.

The RAS/RAF/MEK/ERK pathway regulates proliferation in haematological malignancies and is activated by mutant RAS or RAF, activated receptor tyrosine kinases such as KIT and FLT3, chromosomal translocations such as *BCR-ABL1* or *ETV6-PDGFR*, or chemotherapeutic agents.^6^ Mutations in NRAS, KRAS, and the protein phosphatase PTPN11 are associated not only with relapse and poorer outcomes in childhood leukemia but also with increased sensitivity to MEK inhibitors (MEKi).^7, 8^ Critically, this pathway regulates survival and apoptosis through ERK-mediated phosphorylation of apoptotic effectors such as BAD and BIM or transcriptional regulation of BCL-2 family genes.^6, 9, 10^

In this study, we investigated how MEK/ERK signaling regulates B-ALL proliferation and survival. We found that inhibition of this pathway primed B-ALL cells for death by BCL-2/BCL-XL inhibitors (BCL-2i) through a mechanism dependent on the pro-apoptotic protein BIM. Thus we reveal a synergistic interaction between two pro-survival pathways that has therapeutic potential in a range of B-ALL subtypes.

## Results

### MEK inhibition does not block B-ALL cell growth

**Figure 1:**
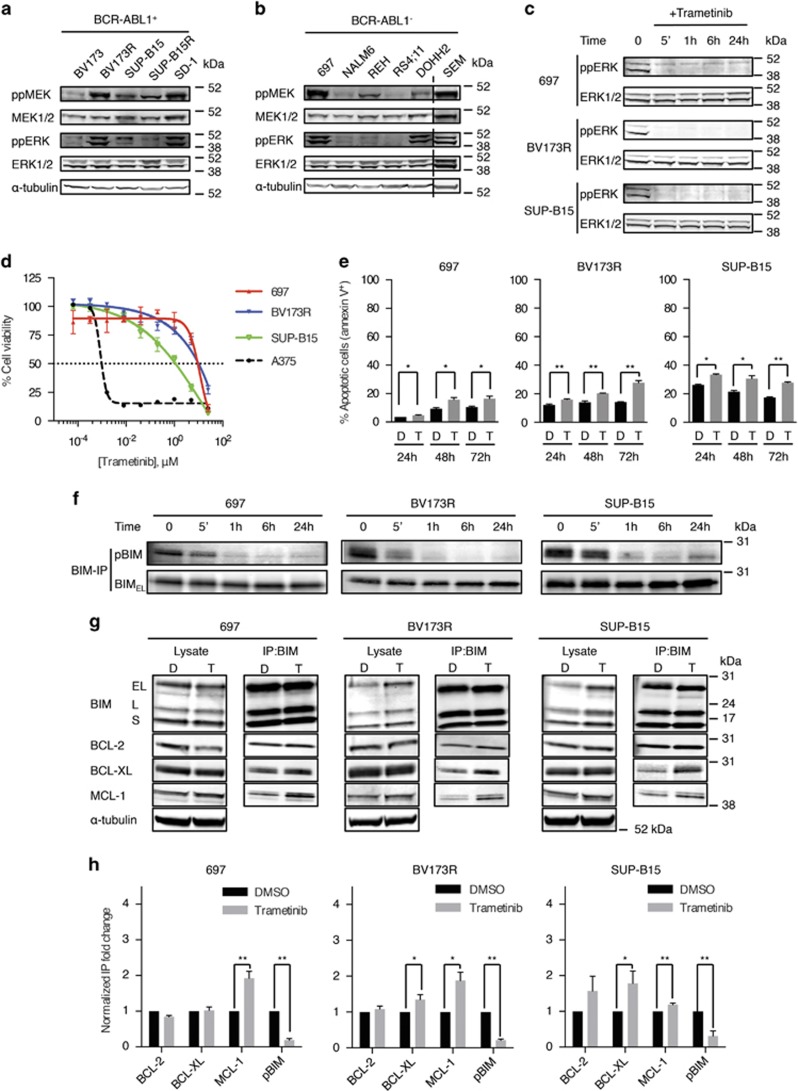
Trametinib primes B-ALL cells to apoptosis through BIM dephosphorylation. (**a** and **b**) Western blottings showing phospho-MEK (ppMEK), total MEK (MEK1/2), phospho-ERK (ppERK), total ERK (ERK1/2), and *α*-tubulin (loading control) in five BCR-ABL1^+^ B-ALL cell lines (**a**) and six BCR-ABL1^−^ cell lines (**b**). The dotted line indicates where discontinuous sections of blots were joined. (**c**) Western blottings showing ppERK and ERK1/2 (loading control) in B-ALL cells treated with trametinib (40 nM) at the indicated times. (**d**) Dose–response curves for trametinib (72 h) treatment of 697, BV173R, and SUP-B15 B-ALL cells. Cell viability (%) is relative to dimethyl sulfoxide (DMSO) control and A375 (BRAF^V600E^ melanoma) cells provide a positive control for sensitive cells. (**e**) Graphs represent apoptotic cells (%) after treatment with DMSO (control) or trametinib (40nM) at the indicated times. (**f**) Western blottings showing phospho-S69-BIM (pBIM) and total BIM (BIM_EL_) in BIM immunoprecipitates from 697, BV173R, and SUP-B15 cells treated with trametinib (40 nM) for the times indicated. (**g**) Western blottings showing BIM, BCL-2, BCL-XL, MCL-1, and *α*-tubulin (loading control) in cell lysates and BIM co-IPs from 697, BV173R, and SUP-B15 cells treated with DMSO (control; D) or trametinib (40 nM; T) for 24 h. (**h**) Graphs represent normalized quantification of BIM co-IPs from triplicate experiments for samples shown in panel (**g**). Error bars in panels (**d**, **e**, and **h**): S.E.M. *P<0.05; **P<0.01

The MEK/ERK pathway is activated downstream of driver oncogenes such as BCR-ABL1 and NRAS,^6^ and accordingly, we observed different levels of MEK/ERK pathway activation not only in six B-ALL cell lines expressing BCR-ABL1^+^ or NRAS^G12D^ but also in four cell lines driven by other oncoproteins, including ETV6-PDGFRB, ETV6-RUNX1, and MLL-AF4, except RS4;11 cells, which demonstrated no MEK/ERK pathway activity (Figures 1a and b; Supplementary Figures S1a and b; Supplementary Table S1). We therefore investigated this pathway in B-ALL cell proliferation and survival. Surprisingly, even profound pathway inhibition by the small-molecule MEKi trametinib (Figure 1c) did not inhibit B-ALL cell growth (Figure 1d; Supplementary Figures S1c and d; Supplementary Table S2) and induced only modest apoptosis (Figure 1e).

### BCL-2 and BCL-XL attenuate the effects of MEK inhibition in B-ALL cells

Thus the MEK/ERK pathway was active in B-ALL cells but was not required for survival, so to investigate the mechanisms underlying these cells' intrinsic resistance to MEKi, we examined BIM and BAD, because ERK phosphorylates these pro-apoptotic proteins, inhibiting their binding to the pro-survival BCL-2 family members and promoting cell survival.^9, 10^ Trametinib did not block BAD phosphorylation on serine 75 (S75) in B-ALL cells (Supplementary Figure S2a), did not change BAD binding to BCL-2 or BCL-XL, and did not induce BAD binding to a third pro-survival protein, MCL-1 (Supplementary Figure S2b). In contrast, trametinib induced robust BIM dephosphorylation on S69 (Figure 1f), and although this did not affect BIM binding to BCL-2, it significantly increased BIM binding to MCL-1 in 697, BV173R, and SUP-B15 cells and to BCL-XL in BV173R and SUP-B15 cells (Figures 1g and h).

**Figure 2:**
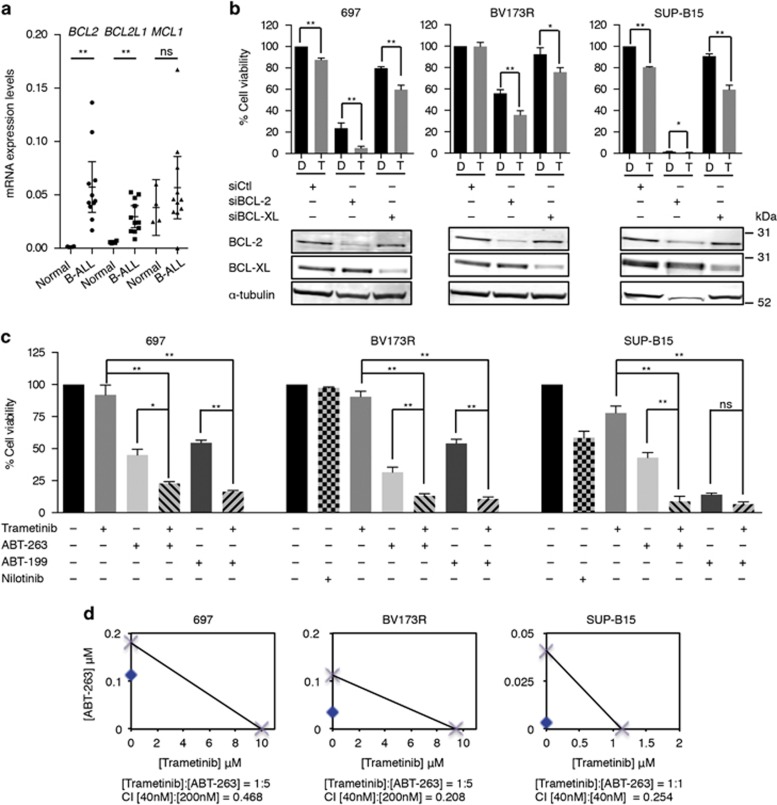
MEKi and BCL-2i synergize to kill B-ALL cells. (**a**) Scatter dot plot showing mRNA expression for *BCL2*, *BCLX (BCL2L1)*, and *MCL1* relative to housekeeping gene control in the 11 B-ALL cell lines (Supplementary Table S1) and normal primary CD34^+^ cells. Error bars: mean with 95% confidence intervals. **P<0.01; NS, not significant. (**b**) Graphs showing cell viability (%) in 697, BV173R, and SUP-B15 cells transfected with control siRNA (siCtl), BCL-2 (siBCL-2), or BCL-XL (siBCL-XL) siRNAs and treated with dimethyl sulfoxide (DMSO; control; D) or trametinib (40nM; T) for 72 h. The western blottings below the graphs show knockdown efficacy. (**c**) Graphs showing cell viability after 72 h at 200 nM (697, BV173R cells) or 40 nM (SUP-B15 cells) ABT-263 or ABT-199 with or without 40 nM trametinib as indicated. BCR-ABL1^+^ cells were also treated with nilotinib (1 *μ*M). Results are relative cell viability (%) to DMSO controls. Error bars in panels (**b** and **c**): S.E.M. *P<0.05; **P<0.01. (**d**) Isobolograms for trametinib/ABT-263 combinations in 697, BV173R, and SUP-B15 cells. Crosses on *x* and *y* axes indicate the IC50 values for each compound. Blue dots show the concentrations of the single drugs that lead to 50% inhibition in cell viability for the given combination ratios. Combination indices (CI) for the combination drug concentrations in panel (**c**) are also indicated (CI<1=synergism)

We were intrigued that, despite these changes, MEK inhibition did not induce profound apoptosis but noted that, compared with normal CD34^+^ hematopoietic cells, *BCL2* and *BCLX (BCL2L1)* were significantly upregulated in B-ALL cells (Figure 2a). Accordingly, BCL-2 depletion significantly reduced B-ALL cell survival, and BCL-XL depletion had a modest effect (Figure 2b). More importantly, trametinib cooperated with BCL-2 or BCL-XL depletion to further suppress viability in these cells (Figure 2b).

### MEKi and BCL-2i cooperate to induce B-ALL cell death

The data above implicated BCL-2 and BCL-XL in intrinsic resistance to MEKi, so we tested whether BCL-2i cooperated with MEKi to suppress B-ALL cell viability. UMI-77, a selective MCL-1 inhibitor did not reduce B-ALL cell viability either alone or in combination with trametinib (Supplementary Table S3; Supplementary Figure S3a). AT-101, which binds to BCL-2 and BCL-XL at 300–400 nM, also failed to reduce B-ALL cell viability alone or in combination with trametinib (Supplementary Table S3; Supplementary Figure S3b). Similarly, sabutoclax, which binds to BCL-2 and BCL-XL at ~300 nM reduced viability modestly by itself but failed to cooperate with trametinib to kill the cells (Supplementary Table S3; Supplementary Figure S3c).

In contrast, ABT-263,^11^ which binds to BCL-2 at 1 nM and BCL-XL at 0.5 nM (Supplementary Table S3), not only inhibited the growth of all three cell lines by itself but also synergized with trametinib to further inhibit cell growth (Figures 2c and d). Similarly, ABT-199,^12^ which binds to BCL-2 at 0.01 nM and BCL-XL at 48 nM (Supplementary Table S3), inhibited cell growth alone, and it cooperated with trametinib to further reduce cell viability (Figure 2c). Note that trametinib/ABT-263 and trametinib/ABT-199 combinations were more effective at reducing cell viability than the TKI nilotinib in BCR-ABL1^+^ cells (Figure 2c). Furthermore, the loss of cell viability with ABT-263 and ABT-199 was linked to increased apoptosis, and these drugs cooperated with trametinib to significantly increase apoptosis in these cells (Supplementary Figure S4a). The death induced by the trametinib/ABT-263 combination was accompanied by loss of mitochondrial membrane potential, demonstrating that apoptosis was mitochondrially mediated (Supplementary Figure S4b). We conclude that trametinib cooperated with the potent BCL-2i ABT-199 and ABT-263 to induce B-ALL cell death.

### BIM mediates synergistic killing of B-ALL cells by MEKi and BCL-2i

**Figure 3:**
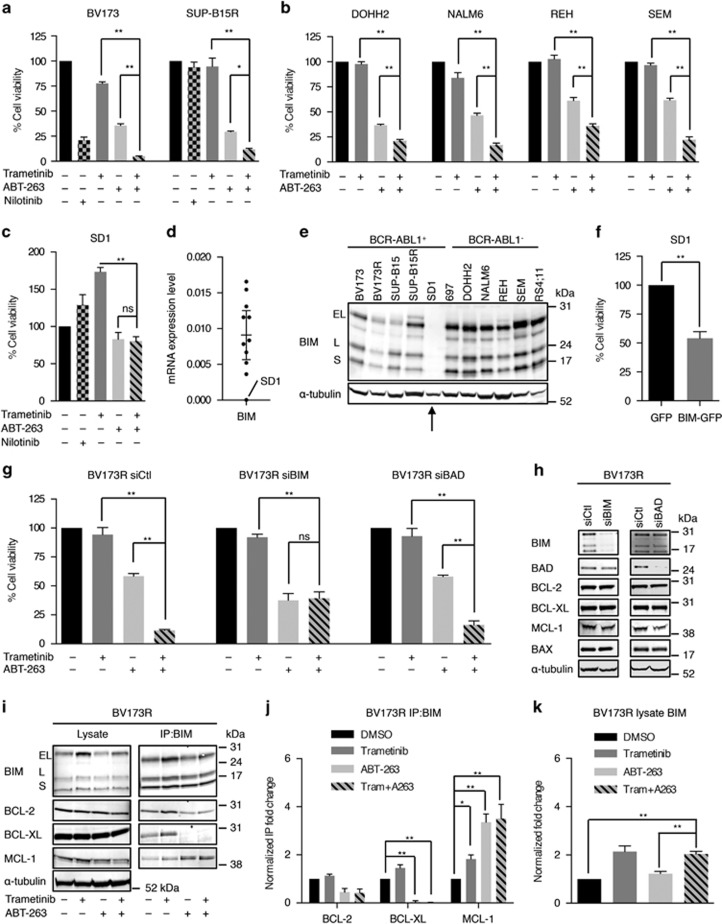
BIM mediates the synergism between trametinib and ABT-263. (**a**) Graphs showing cell viability after 72 h at 200 nM ABT-263 with or without 40 nM trametinib in BCR-ABL1^+^ cells (BV173, SUP-B15R) as indicated. Nilotinib is used at 1 *μ*M. (**b**) Graphs showing cell viability after 72 h at 200 nM (DOHH2, REH, SEM cells) or 1 *μ*M (NALM6 cells) ABT-263 with or without 40 nM trametinib as indicated. (**c**) Graph represents viability of SD1 cells after 72 h at 1 *μ*M ABT-263 with or without 40 nM trametinib as indicated. Nilotinib is used at 1 *μ*M. (**d**) Scatter dot plot showing *BIM (BCL2L11)* mRNA expression relative to housekeeping gene control in the 11 B-ALL cell lines. Error bar: mean with 95% confidence intervals. (**e**) Western blottings show BIM and *α*-tubulin (loading control) expression in the B-ALL cells. (**f**) Graph displays (%) cell viability of SD1 cells 72 h after BIM-GFP transfection relative to GFP-transfected cells. The analysis was performed on cells selected for GFP expression by fluorescence-activated cell sorting. (**g**) Graphs showing BV173R cell viability 72 h after transfection with control siRNA (siCtl), siRNA BIM (siBIM), or siRNA BAD (siBAD) siRNAs and treated with trametinib (40 nM) and/or ABT-263 (200 nM). (**h**) Western blottings showing the level of pro-survival and antisurvival proteins in BV173R cells after transfection with siCtl, siBIM, or siBAD. (**i**) Western blottings showing BIM, BCL-2, BCL-XL, MCL-1, and *α*-tubulin (loading control) in BV173R cell lysates or BIM co-IPs 24 h after treatment with trametinib (40 nM) and/or ABT-263 (200 nM). (**j**) Graphs represent normalized quantification of BIM co-IPs from triplicate experiments for samples shown in panel (**h**). (**k**) Graph shows normalized quantification of total BIM in lysates from triplicate experiments for samples shown in panel (**h**). Results in panels (**a, b, c**, and **g**) are relative cell viability (%) to dimethyl sulfoxide (DMSO) control. Error bars in panels (**a, b, c, f, g, j**, and **k**): S.E.M. *P<0.05; **P<0.01; NS, not significant

We extended our findings to other B-ALL cell lines and found that ABT-263 reduced viability of these cells alone and synergized with trametinib to further suppress viability of BV173, SUP-B15R, DOHH2, NALM6, REH, and SEM cells (Figures 3a and b; Supplementary Figure S5; Supplementary Table S4), and we observed similar results with the ABT-199/trametinib combination (Supplementary Figures S6a–d; Supplementary Table S4). Overall, the trametinib/ABT-263 combination was more effective than single agents in 9/11 lines and the trametinib/ABT-199 combination was more effective than single agents in 6/11 lines, so we were intrigued that the combinations did not synergize to inhibit the growth of RS4;11 and SD1 cells (Figure 3c; Supplementary Figures S6c and e). As shown above, the MEK/ERK pathway is not active in RS4;11 cells (Supplementary Figures S1a and b) and this is a prerequisite for the cooperation between MEKi and BCL2i. However, SD1 cells presented high levels of MEK/ERK activity (Figure 1a) but intriguingly did not express BIM (Figures 3d and e) and BIM re-expression was sufficient to kill these cells (Figure 3f). This implicated BIM in the synergistic killing of B-ALL cells by trametinib and ABT-263, and accordingly, we show that when BIM was depleted, trametinib no longer synergized with ABT-263 to kill BV173R or 697 cells (Figures 3g and h; Supplementary Figures S7a and b).

These data showed that BIM mediated the synergistic killing of B-ALL cells by MEKi and BCL-2i, so we investigated the mechanism. Our data above showed that trametinib increased BIM binding to MCL-1 (Figures 1g and h), and we found that ABT-263 alone or in combination with trametinib further increased BIM binding to MCL-1 (Figures 3i and j; Supplementary Figures S7c and d). Moreover, we observed increased BIM levels in the presence of trametinib alone or in combination with ABT-263 (Figure 3k; Supplementary Figure S7e), which could be due to the increased stability of the dephosphorylated form.^10^ Importantly, the sensitivity of B-ALL cells to ABT-263 was negatively correlated with endogenous levels of BIM and MCL-1 (Supplementary Figures S8a and b), and when MCL-1 was depleted, ABT-263 strongly inhibited B-ALL cell proliferation even in the absence of trametinib (Supplementary Figures S8c and d). This suggested that the increase in BIM protein and its interaction with MCL-1 in the presence of both MEKi and BCL-2i, neutralized MCL-1, a mechanism that explains the synergistic effect observed between trametinib and ABT-263 in these cells.

### MEKi and BCL-2i cooperate to kill primary B-ALL cells and to delay the onset of B-ALL *in vivo*

**Figure 4:**
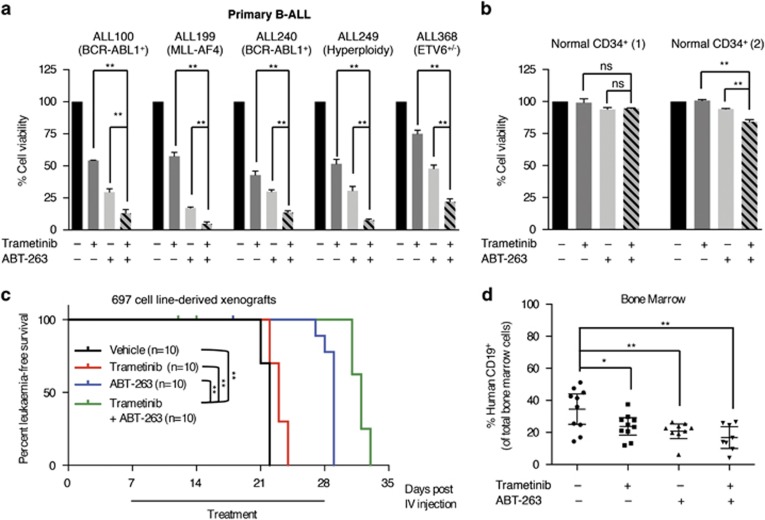
Trametinib and ABT-263 cooperate to kill primary B-ALL cells and delay the onset of leukemia *in vivo*. (**a**) Graphs showing viability of purified CD34^+^ B-ALL cells from five patients 72 h after treatment with trametinib (40 nM) and/or ABT-263 (40 nM). (**b**) Graphs showing viability of normal CD34^+^ cells purified from two non-leukemic individuals 72 h after treatment with trametinib (40 nM) and/or ABT-263 (40 nM). Results are shown as percentage of cell viability relative to dimethyl sulfoxide control. Error bars: S.E.M. (**c**) Kaplan–Meier survival curves showing leukemia-free survival of mice inoculated with 697 cells and treated with vehicle, trametinib (0.15 mg/Kg), ABT-263 (100 mg/Kg), or trametinib (0.15 mg/Kg) plus ABT-263 (100 mg/Kg) for 3 weeks. (**d**) Scatter dot plot showing human CD19^+^ cells (%) measured by flow cytometry in the bone marrow of the mice at death in each treatment arm shown in panel (**c**). Error bars: mean with 95% confidence intervals. *P<0.05; **P<0.01; NS, not significant

Next we investigated whether MEKi and BCL-2i combinations could also kill freshly isolated cells from B-ALL patients (Supplementary Table S5, Figure 4a). At clinically achievable doses, trametinib and ABT-263 reduced the viability of undifferentiated CD34^+^/CD19^hi^ cells purified from peripheral blood mononucleated cells of five B-ALL patients, but more importantly, these agents cooperated to further reduce cell viability in these cells (Figure 4a). As a control, we showed that trametinib and ABT-263 had a negligible effect on normal CD34^+^ cell viability from two non-ALL individuals (Figure 4b), showing that this combination specifically targeted the leukemic cells while sparing normal hematopoietic cells.

Finally, 697 cells were inoculated into non-obese diabetic *scid* gamma (NSG) mice, and the mice were treated with trametinib, ABT-263, or the combination for 3 weeks. Mice were killed at the onset of leukemia, manifested by ill health. We observed a small increase in survival with trametinib and a more substantial survival advantage with ABT-263, but critically these compounds cooperated to give a significant if modest increase in survival compared with the single-agent treatments (Figure 4c). Furthermore, trametinib and ABT-263 alone reduced the bone marrow leukemic cell burden by ~40% (*P*<0.05) compared with controls, but together, these agents reduced the leukemic cell burden in the bone marrow by ~60% (*P*<0.001; Figure 4d).

## Discussion

This study demonstrates that MEKi plus BCL-2i is a promising drug combination in B-ALL cells. Recent studies suggested that only leukemias with mutations in RAS could benefit from MEKi therapy,^7, 13^ but we showed that this pathway was activated in B-ALL cell lines driven by a range of genetic aberrations. We showed that inhibition of ERK resulted in dephosphorylation of the pro-apoptotic protein BIM_EL_, an important regulator of apoptosis in normal and malignant B cells, and a tumor suppressor in B-cell malignancies.^14^ It has been reported that BIM phosphorylation by ERK1/2 increased cell survival through reduced BIM binding to pro-survival proteins, including BCL-XL and MCL-1, and by increased BIM degradation.^10, 15^ Accordingly, we showed that trametinib caused BIM dephosphorylation, resulting in upregulation of BIM protein and its binding to MCL-1, consistent with a recent study in chronic lymphocytic leukemia (CLL) showing that CLL cells were MCL-1-dependent and phosphorylated BIM was unable to interact with MCL-1 to induce apoptosis.^16^

However, despite priming B-ALL cells for apoptosis, trametinib only modestly affected their viability, suggesting that BIM dephosphorylation alone was insufficient to overcome pro-survival signals. Accordingly, the pro-survival genes *BCL2* and *BCLX* were elevated in B-ALL cells compared with normal CD34^+^ cells. Upregulation of antiapoptotic proteins has been reported in some hematological malignancies,^17, 18^ stimulating the development of potent BCL-2i.^11, 12^ Importantly, it was reported that BCL-2 overexpression in lymphoid malignancies inhibited apoptosis through BIM sequestration^19, 20^ and that BCL-2i induced apoptosis by disrupting the BCL-2/BIM complex.

Here we showed that B-ALL cells demonstrate a range of sensitivities to ABT-199 and ABT-263, although complete loss of cell viability was not seen at clinically achievable doses. However, the cells could be killed when BCL-2i were combined with MEKi or when MCL-1 was depleted. Thus, in accordance with previous studies,^21, 22^ we showed that intrinsic resistance of B-ALL cells to BCL-2i was mediated by MCL-1 and that the pro-survival effects of MCL-1 were overcome by BIM, which is phosphorylated and inactivated downstream of MEK/ERK. Previous studies showed that MEKi and BCL-2i cooperated to inhibit the growth of KRAS and BRAF mutant solid tumors.^23, 24^ In those cases, MEK appeared to be the primary growth inhibitor, and BCL-2i enhanced the effect. However, we showed here that MEKi had a modest effect in B-ALL cells, but MEK inhibition sensitized the cells to BCL-2i through a MEK/ERK signaling-dependent mechanism mediated by BIM.

Thus we posit that the mechanism by which MEKi and BCL-2i cooperated to kill B-ALL cells was that MEK inhibition caused BIM dephosphorylation and upregulation at the protein level, allowing BIM to bind to and neutralize MCL-1, removing one survival signal. The concomitant inhibition of BCL-2 and BCL-XL removed the other survival signals, leading to cell death. Importantly, this cooperative effect occurred in B-ALL cells driven by a range of genetic abnormalities. We validated our findings in primary CD34^+^CD19^hi^ B-ALL cells at clinically achievable doses for both drugs while demonstrating that the combination spared normal CD34^+^ cells. Additionally, as the effect was mediated by BIM, the expression of this protein could serve as a convenient biomarker to stratify patients for this combination therapy. Our findings provide further insights into B-ALL cell biology and survival mechanisms and identify MEK and BCL-2/BCL-XL as targets that could be exploited for effective management of this disease.

## Materials and Methods

### Cell culture

For cell lines and their driver oncogenes, see Supplementary Table S1. BV173, BV173R, 697, and SD1 cells were cultured in RPMI 1640 medium supplemented with 20% fetal bovine serum (FBS) and 1% penicillin/streptomycin (Life Technologies, Paisley, UK). DOHH2, NALM6, REH, and SEM cells were cultured in RPMI 1640 medium supplemented with 10% FBS and 1% penicillin/streptomycin. SUP-B15 and SUP-B15R cells were cultured in McCoy's 5A medium (Life Technologies) supplemented with 20% FBS and 1% penicillin/streptomycin. RS4;11 cells cultured in α-MEM medium (Life Technologies) supplemented with 10% FBS and 1% penicillin/streptomycin.

Primary CD34^+^ ALL and normal cells were cultured in IMDM medium (Life Technologies) supplemented with 25% BIT (bovine serum albumin/insulin/transferrin; StemCell Technologies, Vancouver, BC, Canada), L-glutamine, penicillin/streptomycin, and 125 *μ*M 2-mercaptoethanol (Life Technologies). ALL CD34^+^ cells were cultured in the presence of FLT3-ligand (100 ng/ml), IL-7 (100 ng/ml), and SCF (100 ng/ml), as described.^25^ Normal CD34^+^ cells were cultured in a five growth factor cocktail containing FLT3-ligand (100 ng/ml), G-CSF (20 ng/ml), IL-3 (20 ng/ml), IL-6 (20 ng/ml), and SCF (100 ng/ml) (all growth factors are from PeproTech, Rocky Hill, NJ, USA).

### Primary cells from patient samples

Peripheral blood from B-ALL patients was collected at diagnosis following informed consent and ethical approval from the scientific sub-committee of the Manchester Cancer Research Centre Tissue Biobank (07/H1003/161+5) and in compliance with the ethical and legal framework of the Declaration of Helsinki and the UK's Human Tissue Act, 2004. Normal mobilized CD34^+^ cells surplus to requirements were from patients undergoing chemotherapy and autologous transplantation, and their use was authorized by the Salford and Trafford Research Ethics Committee (08/H1004/114) for samples collected since 2006 and following written informed consent of donors. Mononuclear cells were isolated from peripheral blood by Ficoll separation. CD34^+^ cell enrichment was by AutoMACS magnetic column separation (Miltenyi Biotec, Bergisch Gladbach, Germany) using the CD34 Human Microbead Kits (Miltenyi Biotec). CD34^+^ cells from B-ALL samples were >90% CD19^+^ by flow cytometry (anti-human CD19 PE, eBioscience, San Diego, CA, USA). For clinical information and genetic abnormalities in patient samples, see Supplementary Table S5.

### Reagents (inhibitors, siRNAs, and plasmids)

Stock solutions (10 mM) of trametinib, ABT-199, ABT-263, AT-101, sabutoclax, and UMI-77 (Selleckchem, Houston, TX, USA) were prepared in DMSO and stored at −80 °C. Appropriate concentrations of each drug were prepared in DMSO prior to use. Cell lines were transfected with siRNAs to BCL-2 (ON-TARGETplus Human BCL2 SMARTpool; Dharmacon, Lafayette, CO, USA), BCL-XL (Hs_BCL2L1_2, QIAGEN, Hilden, Germany), MCL-1 (Hs_MCL1_6, QIAGEN), BIM (Hs_BCL2L11_5, QIAGEN), and BAD (Hs_BAD_3, QIAGEN) using 4D-Nucleofector system (Lonza, Basel, Switzerland). AllStars Negative Control siRNA (QIAGEN) was the negative control.

The BIM-GFP expression plasmid was constructed by cloning the human BIM_EL_ cDNA (BCL2L11-001, ENST00000393256) into a pMCEF vector and moving the BamHI and AgeI restriction fragment into the pEGFP-N1 vector. The plasmid was transfected into SD1 cells using 4D-Nucleofector system (Lonza) and GFP^+^ cells were sorted by flow cytometry after 48 h.

### Immunoprecipitation and immunoblot assays

Cell lysates were prepared using cell lysis buffer (20 mM Tris-HCl (pH 7.5), 150 mM NaCl, 1 mM Na_2_EDTA, 1mM EGTA, 1% Triton, 2.5 mM sodium pyrophosphate, 1 mM beta-glycerophosphate, 1mM Na_3_VO_4_, 1 *μ*g/ml leupeptin; Cell Signaling, Danvers, MA, USA) with protease inhibitor cocktail and phosphatase inhibitor cocktail 3 (Sigma Aldrich, St. Louis, MO, USA). For co-immunoprecipitation (co-IP), cell extracts were prepared in CHAPS lysis buffer (20 mM Tris-HCl (pH 7.5), 137 mM NaCl, 1% CHAPS, 1 mM EDTA, 1mM EGTA, 5 mM MgCl_2_, protease inhibitor cocktail, and phosphatase inhibitor cocktail 3) as described.^26^ Protein extracts were immunoprecipitated with anti-BIM (C34C5; Cell Signaling) and anti-BAD (C-7; Santa Cruz Biotechnology, Dallas, TX, USA) at 4 °C for 2 h. Immunoprecipitates were captured by 50 *μ*l of protein G-agarose beads in CHAPS lysis buffer (Roche, Basel, Switzerland) at 4 °C for 3 h. Immunoprecipitates were recovered by centrifugation, washed three times in CHAPS buffer, and eluted in boiling NuPAGE LDS Sample Buffer and NuPAGE Sample Reducing Agent (Life Technologies). Immunoprecipitates and cell lysates (40 *μ*g of protein) were separated by SDS-PAGE on NuPAGE Bis-Tris gels (Life Technologies). Immunoblots were performed with antibodies from Cell Signaling (BAX, BCL-XL, BIM, phosphoSer69-BIM, ERK1/2, MEK1/2, and phosphoSer217/Ser221-MEK), Santa Cruz (BCL-2 and BAD), Sigma-Aldrich (MCL-1, diphosphorylated ERK1/2, and α-tubulin), and Abcam (Cambridge, UK; phosphoSer112-BAD), probed by fluorescence secondary antibodies (Li-Cor, Lincoln, NE, USA) and analyzed on an Odyssey CLx infrared imaging system (Li-Cor). Densitometry analyses of western blotting bands were performed on Image Studio Lite (Li-Cor). For BIM co-IP assays, normalized IP fold changes were calculated from band densitometries for BIM and its interacting proteins as follows:


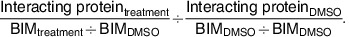


### Cell viability assays

Cell lines were seeded at 1 × 10^4^–2 × 10^4^ cells/well and primary cells were seeded at 5 × 10^4^ cells/well in 96-well plates and treated with DMSO (control) or small-molecule inhibitors for 72 h at 37 °C and 5% CO_2_. Cell proliferation and viability was measured by a methanethiosulfonate-based assay (CellTiter 96 Aqueous One Solution; Promega, Madison, WI, USA). Results were reported as the percentage of viability for cell lines or viability for primary cells relative to DMSO controls, and the S.E.M. was calculated from triplicates. The combined effect of trametinib and ABT-263 in fixed ratio combinations was quantified by the Chou–Talalay method.^27^

### Apoptosis assays

The percentage of apoptotic cells in the presence or absence of small-molecule inhibitors was determined using 7-AAD (7-aminoactinomycin D) and PE annexin V probes (BD Biosciences, Franklin Lakes, NJ, USA) on a BD Accuri C6 flow cytometer (BD Biosciences). Results were reported as the average percentage of annexin V^+^ cells, and the S.E.M. was calculated from triplicates.

Mitochondrial membrane potential was determined 24 h posttreatment with DMSO or small-molecule inhibitors using a TMRE (tetramethylrhodamine, ethyl ester) Mitochondrial Membrane Potential Assay Kit (Abcam) according to the manufacturer's instructions. Cells treated with FCCP (carbonyl cyanide 4-(trifluoromethoxy)phenylhydrazone), an ionophore uncoupler of oxidative phosphorylation, were used as positive control for the loss of mitochondrial membrane potential.

### Real-time PCR (RT-PCR)

RNA was extracted from cells by the RNeasy Mini Kit (QIAGEN), and cDNA was synthesized using the High-Capacity RNA-to-cDNA Kit (Life Technologies), according to the manufacturers' instructions. For mRNA expression levels, RT-PCR assays were performed using human TaqMan probes for *BCL2* (Hs00608023_m1), *BCL2L1 (BCLX)* (Hs00236329_m1), *BCL2L11 (BIM)* (Hs00708019_s1), *MCL1* (Hs01050896_m1), and human *GAPDH* endogenous control probe (4352934E, Life Technologies). Reactions were performed in triplicate on an ABI PRISM 7900HT platform (Life Technologies), and results were reported as the expression levels relative to the housekeeping gene in the same cells.

### Generation of xenografts and *in vivo* drug treatments

All procedures involving animals, carried out under license PPL-70/7701, were performed in accordance with ARRIVE guidelines and National Home Office regulations under the Animals (Scientific Procedures) Act 1986 and reviewed by the Cancer Research UK Manchester Institute's Animal Welfare and Ethics Review Body (AWERB). 697 cell-derived xeongrafts were generated by intravenous injection of 1 × 10^4^ 697 cells into the tail vein of 6–8-week-old female NSG mice (Charles River Laboratories, Wilmington, MA, USA). Seven days postinjection, mice were randomized into four treatment groups of similar total average body weight (*n*=10 mice per group) and vehicle (10% ethanol, 30% polyethylene glycol (Sigma-Aldrich), and 60% Phosal 50 PG (Lipoid, Ludwigshafen, Germany)), trametinib (0.15 mg/Kg), ABT-263 (100 mg/Kg), or the combination were administered daily by oral gavage for 3 weeks, as described for ABT-263's *in vivo* dosing protocol.^11^ For combination therapy, trametinib was administered 1–2 h before ABT-263. Mice were killed upon demonstrating ill health (loss of body weight, lack of vitality, hind limb paralysis), and bone marrow cells were extracted for flow cytometric analysis of human CD19^+^ cells.

### Statistics

Data are presented as means±S.E.M., and *P*-values were calculated using unpaired Student's *t*-test for comparisons involving two groups and one-way ANOVA with Holm–Sidak multiple comparison correction tests for comparisons involving more than two groups. Furthermore, two-way ANOVA with Holm–Sidak multiple comparisons correction tests were used for comparisons of fold changes in BIM co-IP assays in four treatment conditions. Survival curves for *in vivo* treatments were compared using Logrank (Mantel–Cox test). *P-*values <0.05 are considered statistically significant.

## Abbreviations

B-ALL: B-cell acute lymphoblastic leukemia
TKI: tyrosine kinase inhibitor
MEKi: MEK inhibitor
BCL-2i: BCL-2 inhibitor
NSG: non-obese diabetic *scid* gamma
CLL: chronic lymphocytic leukemia
FBS: fetal bovine serum
co-IP: co-immunoprecipitation

## Supplementary Material

Supplementary Informations
