# Supplementary material for: BIM mediates synergistic killing of B-cell acute lymphoblastic leukemia cells by BCL-2 and MEK inhibitors
Source: Cell Death Dis. 2016 Apr 7;7(4):e2177–. doi: 10.1038/cddis.2016.70 (PMC4855656; doi:10.1038/cddis.2016.70)
Supplement: Supplementary Informations [file cddis201670x1.docx]

**Supplementary Information**

**BIM mediates synergistic killing of B-cell acute lymphoblastic leukemia cells by BCL-2 and MEK inhibitors**

Koorosh Korfi, Matthew Smith, Jacqueline Swan, Tim C P Somervaille, Nathalie Dhomen and Richard Marais.

**Inventory of Supplementary Information**

**Supplementary Figure S1.** MEK inhibition does not inhibit B-ALL cell growth.

**Supplementary Figure S2.** BAD interaction with pro-survival proteins is not affected by MEK inhibition.

**Supplementary Figure S3.** Low potency BCL-2 family inhibitors do not synergize with trametinib to inhibit B-ALL cell growth.

**Supplementary Figure S4.** Trametinib/ABT-263 combination cooperatively induces apoptosis in B-ALL cells.

**Supplementary Figure S5.** Trametinib/ABT-263 combination effect in B-ALL cells is synergistic.

**Supplementary Figure S6.** Trametinib and ABT-199 cooperate to kill B-ALL cells.

**Supplementary Figure S7.** BIM mediates synergistic killing of 697 cells by trametinib/ABT-263 combination.

**Supplementary Figure S8.** B-ALL cell sensitivity to ABT-263 is negatively correlated with BIM and MCL-1 levels.

**Supplementary Table S1.** List of cell lines used in this study and their genetic composition.

**Supplementary Table S2.** B-ALL cell lines IC_50_s for trametinib.

**Supplementary Table S3.** Inhibition constants of BCL-2 family inhibitors.

**Supplementary Table S4.** B-ALL cell lines IC_50_s for ABT199 and ABT-263.

**Supplementary Table S5.** List of patient samples used in this study and their genetic abnormalities.

**References**

**
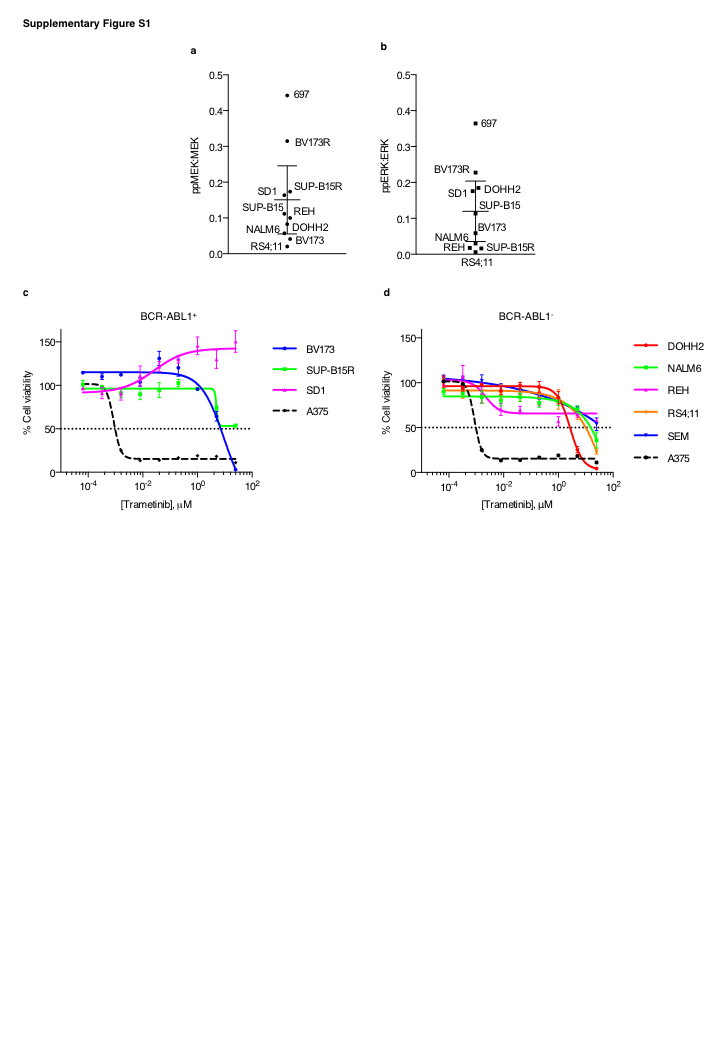
**

**Supplementary Figure S1. MEK inhibition does not inhibit B-ALL cell growth. (a, b)** Scatter dot plot showing ppMEK:MEK (a) and ppERK:ERK (b) ratios obtained from the western blot quantifications of the 11 B-ALL cell lines shown in Figures 1a and b. Error bars: mean with 95% confidence intervals. **(c)** Dose response curves of trametinib treatment for BCR-ABL1^+^ (BV173, SUP-B15R, SD1) B-ALL, and **(d)** BCR-ABL1^-^ (DOHH2, NALM6, REH, RS4;11, SEM) B-ALL cells 72 hours after drug addition. Cell viability (%) is relative to DMSO control. A375 (BRAF^V600E^ melanoma) cells are included as a positive control for sensitive cells. Half maximal inhibitory concentrations (IC_50_s) for all cell lines are shown in Supplementary Table S2.

**
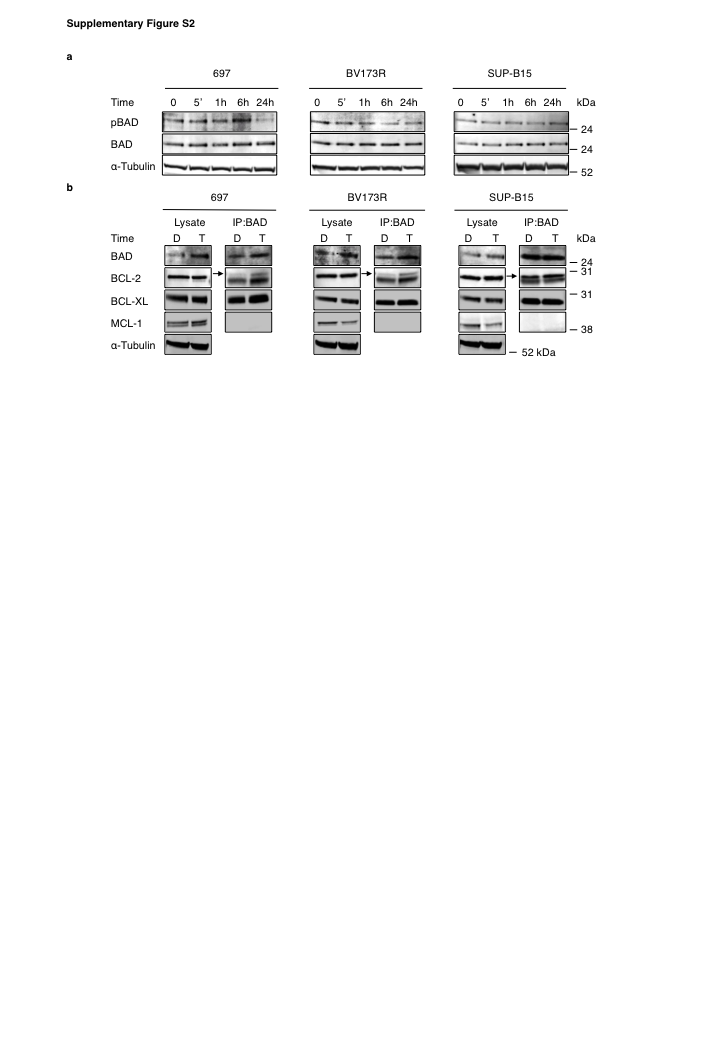
**

**Supplementary Figure S2. BAD interaction with pro-survival proteins is not affected by MEK inhibition. (a)** Western blots for phosphoS75-BAD (pBAD), BAD, and α-tubulin (loading control) in lysates from cells treated with trametinib (40nM) for the times indicated. **(b)** Western blots showing BAD, BCL-2, BCL-XL, MCL-1, and α-tubulin (loading control) in cell lysates and BAD immunoprecipitates from 697, BV173R and SUP-B15 cells treated with DMSO (control; D) or trametinib (40nM; T) for 24 hours. BCL-2 bands in BAD-IP blots are indicated with an arrow.

**
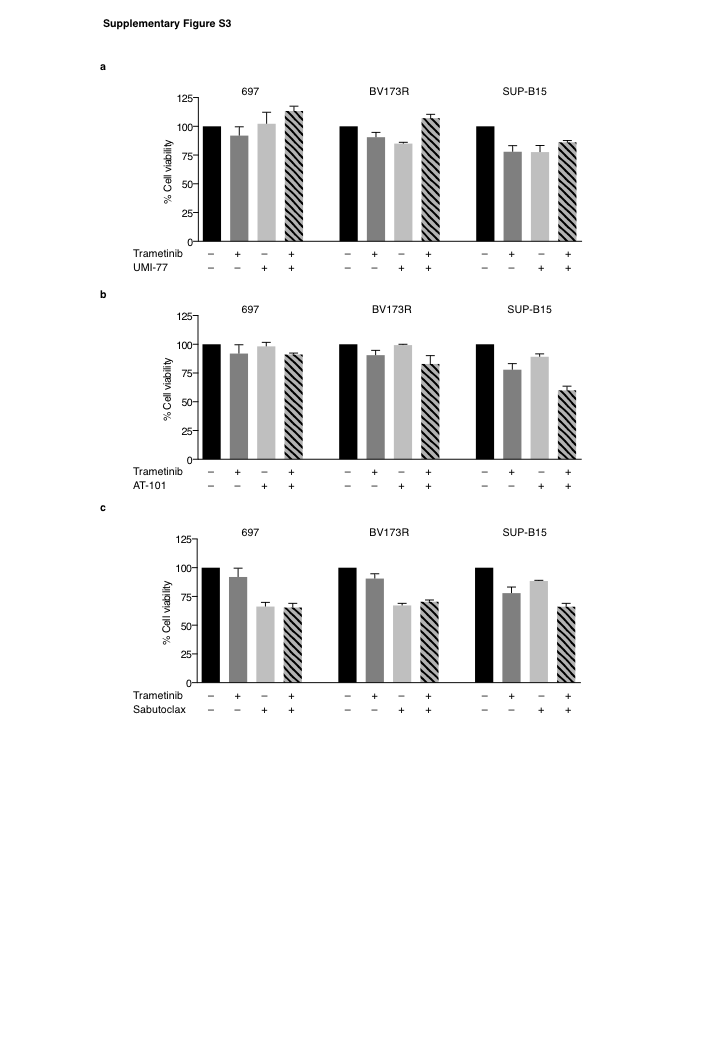
**

**Supplementary Figure S3. Low potency BCL-2 family inhibitors do not cooperate with trametinib to inhibit B-ALL cell growth. (a)** Graphs showing cell viability of 697, BV173R and SUP-B15 cells 72 hours after treatment with UMI-77[^1^](#_ENREF_1) (1μM) with or without trametinib (40nM) as indicated. **(b)** Graphs showing cell viability of 697, BV173R and SUP-B15 cells 72 hours after treatment with AT-101[^2^](#_ENREF_2) (1μM) with or without trametinib (40nM) as indicated. **(c)** Graphs showing cell viability of 697, BV173R and SUP-B15 cells 72 hours after treatment with sabutoclax[^3^](#_ENREF_3) (1μM) with or without trametinib (40nM) as indicated. Results in a, b, and c are relative (%) cell viability to DMSO controls. Error bars: standard error of mean.


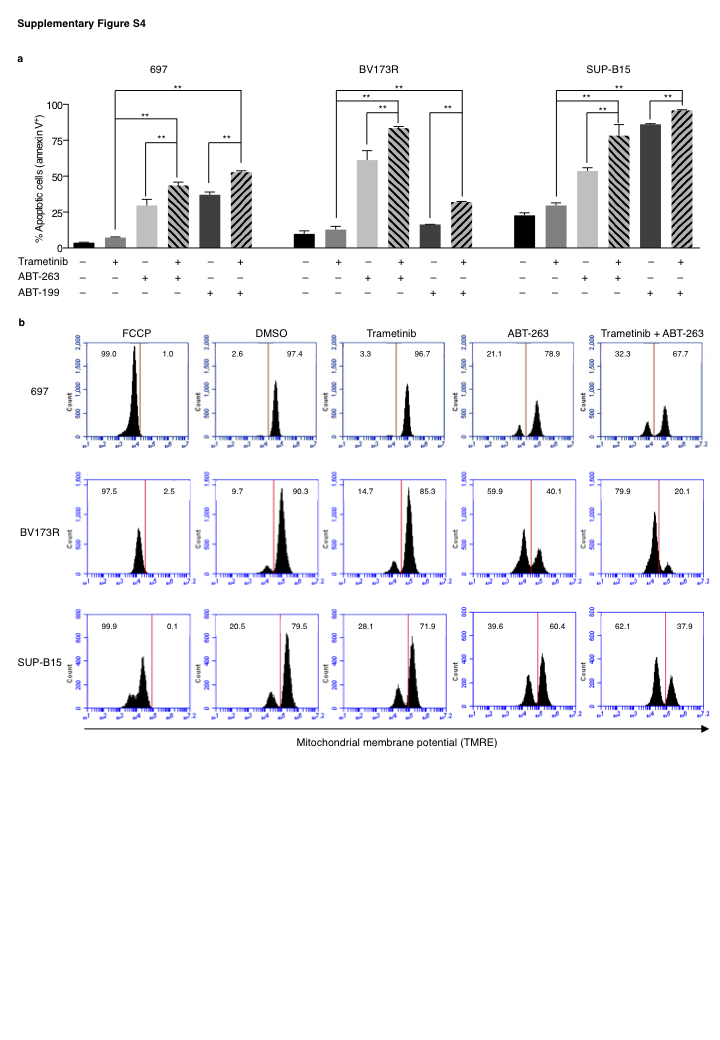


**Supplementary Figure S4. Trametinib/ABT-263 combination cooperatively induces apoptosis in B-ALL cells. (a)** Graph showing apoptotic cells (% annexin V^+^ cells) after 48 hours at 200nM (697, BV173R cells) or 40nM (SUP-B15 cells) ABT-263 or ABT-199 with or without 40nM trametinib as indicated. Error bars: standard error of mean. *p<0.05; **p<0.01. **(b)** Histograms showing mitochondrial membrane potential (TMRE signal) after 24 hours at drug concentrations used above (a). Values represent the proportion (%) of TMRE^+^ or TMRE^-^ cells from total number of cells. FCCP-treated cells are positive controls for the loss of mitochondrial potential.

**
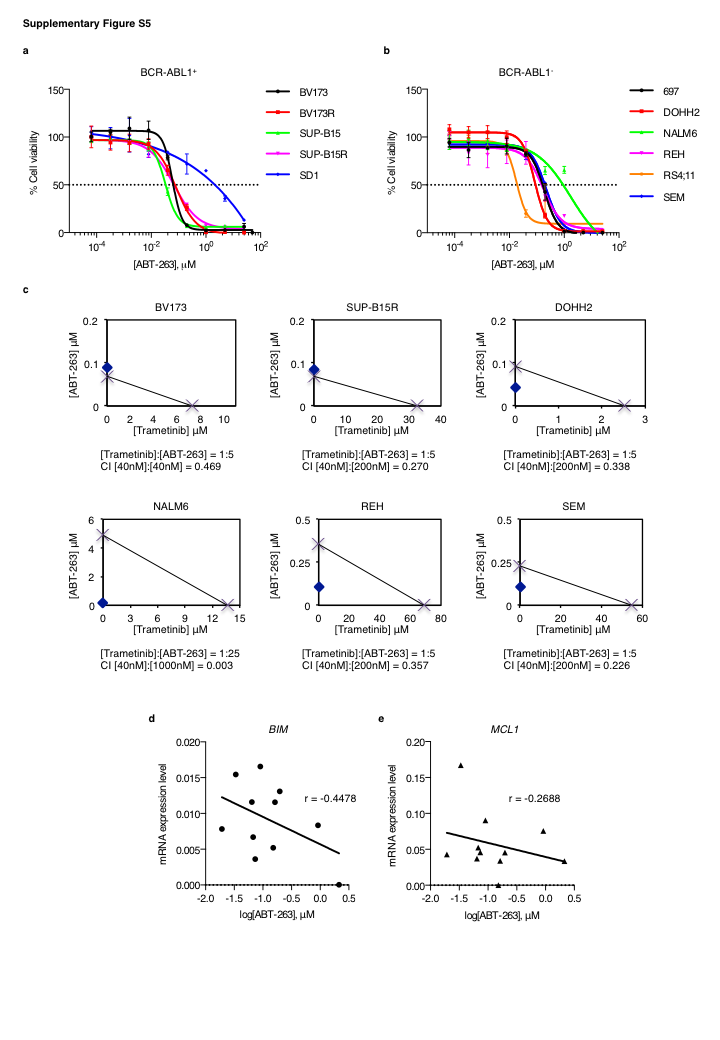
**

**Supplementary Figure S5. Trametinib/ABT-263 combination effect in B-ALL cells is synergistic.** Dose response curves of ABT-263 treatment for **(a)** BCR-ABL1^+^ B-ALL and **(b)** BCR-ABL1^-^ B-ALL cells 72 hours after drug addition. Cell viability (%) is relative to DMSO control. IC_50_s are shown in Supplementary Table S4. **(c)** Isobolograms for trametinib/ABT-263 combinations in BV173, SUP-B15R, DOHH2, NALM6, REH, and SEM cells. Crosses on x- and y-axes indicate the IC50 values for each compound. Blue dots show the concentrations of the single drugs that lead to 50% inhibition in cell viability for the given combination ratios. Combination indices (CI) for the combination drug concentrations shown in Figures 3a and b are also indicated (CI<1 = synergism). Note that in BV173 and SUP-B15R cells synergistic combination was not observed at the entire range of indicated combinations as demonstrated by blue dots above the diagonal line.

**
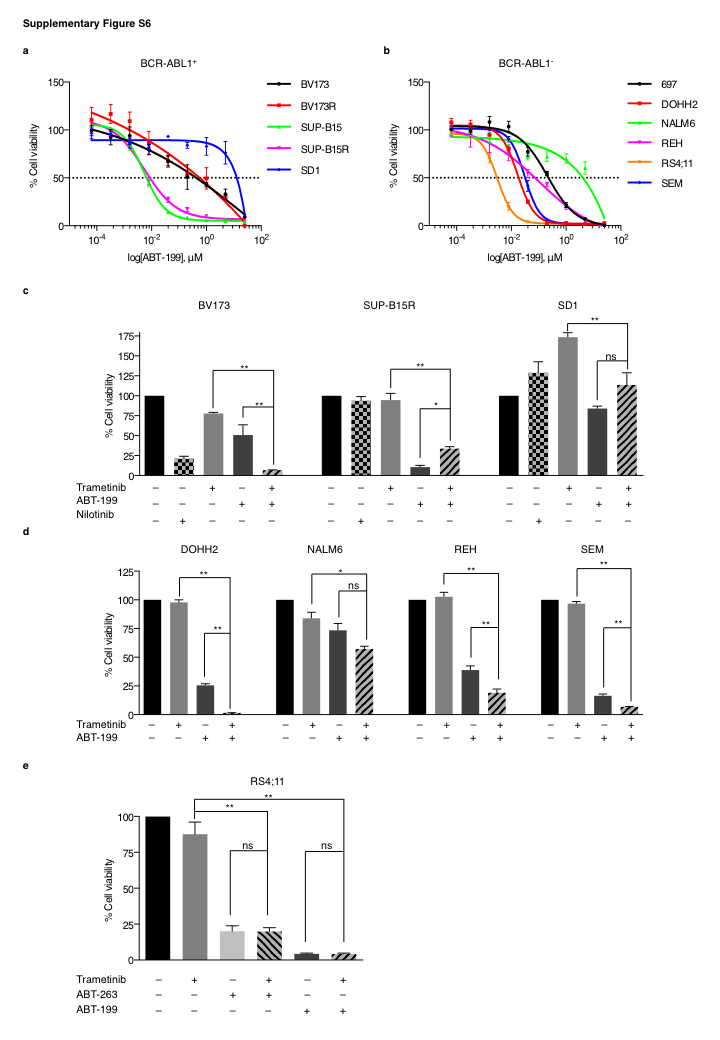
**

**Supplementary Figure S6. Trametinib and ABT-199 cooperate to kill B-ALL cells.** Dose response curves of ABT-199 treatment for **(a)** BCR-ABL1^+^ B-ALL and **(b)** BCR-ABL1^-^ B-ALL cells 72 hours after drug addition. Cell viability (%) is relative to DMSO control. IC_50_s for all cell lines are shown in Supplementary Table S4. **(c)** Graphs showing BCR-ABL1^+^ cell viability after 72 hours at 200nM (BV173, SUP-B15R cells) or 1µM (SD1 cells) ABT-199 with or without 40nM trametinib as indicated. Nilotinib (1μM) treatments are also shown. **(d)** Graphs showing BCR-ABL1^-^ cell viability after 72 hours at 200nM (DOHH2, REH, SEM cells) or 1µM (NALM6 cells) ABT-199 with or without 40nM trametinib as indicated. **(e)** Graph shows RS4;11 cell viability after 72 hours at 40nM ABT-263 or ABT-199 with or without 40nM trametinib as indicated. Results in b-e are relative (%) cell viability to DMSO controls. Error bars: standard error of mean. * p<0.05; **p<0.01; ns, not significant.

**
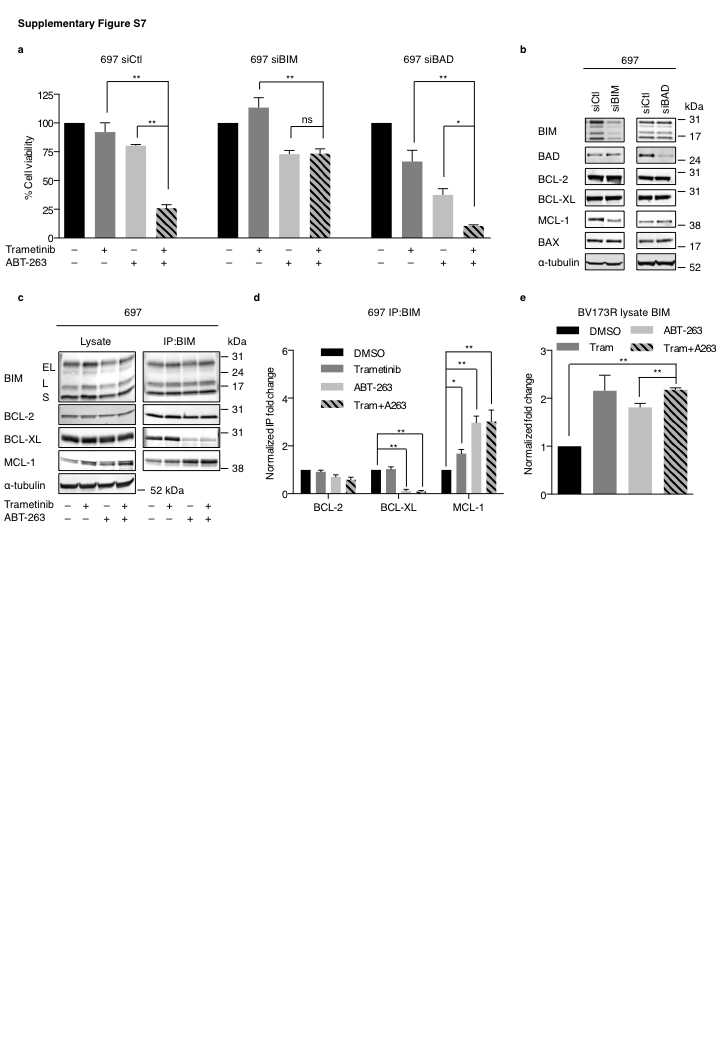
**

**Supplementary Figure S7. BIM mediates synergistic killing of 697 cells by trametinib/ABT-263 combination. (a)** Graphs showing (%) relative 697 cell viability 72 hours after transfection with control (siCtl), BIM (siBIM) or BAD (siBAD) siRNA and treatment with trametinib (40nM) and/or ABT-263 (200nM). Error bars: standard error of mean. **p<0.01; ns, not significant. **(b)** Western blots showing BIM, BAD, BCL-2, BCL-XL, MCL-1, BAX, and α-tubulin (loading control) in 697 cells after transfection with siCtl, siBIM or siBAD siRNAs. **(c)** Western blots showing BIM, BCL-2, BCL-XL, MCL-1, and α-tubulin (loading control) in 697 cell lysates or BIM immunoprecipitates (IP:BIM) 24 hours after treatment with trametinib (40nM) and/or ABT-263 (200nM). **(d)** Graphs showing quantification of BIM immunoprecipitations from triplicate experiments for samples shown in (c) that are normalized against immunoprecipitated levels of BIM. **(e)** Graph shows normalized quantification of total BIM in lysates from triplicate experiments for samples shown in (c). Error bars in d and e: standard error of mean. *p<0.05; **p<0.01.


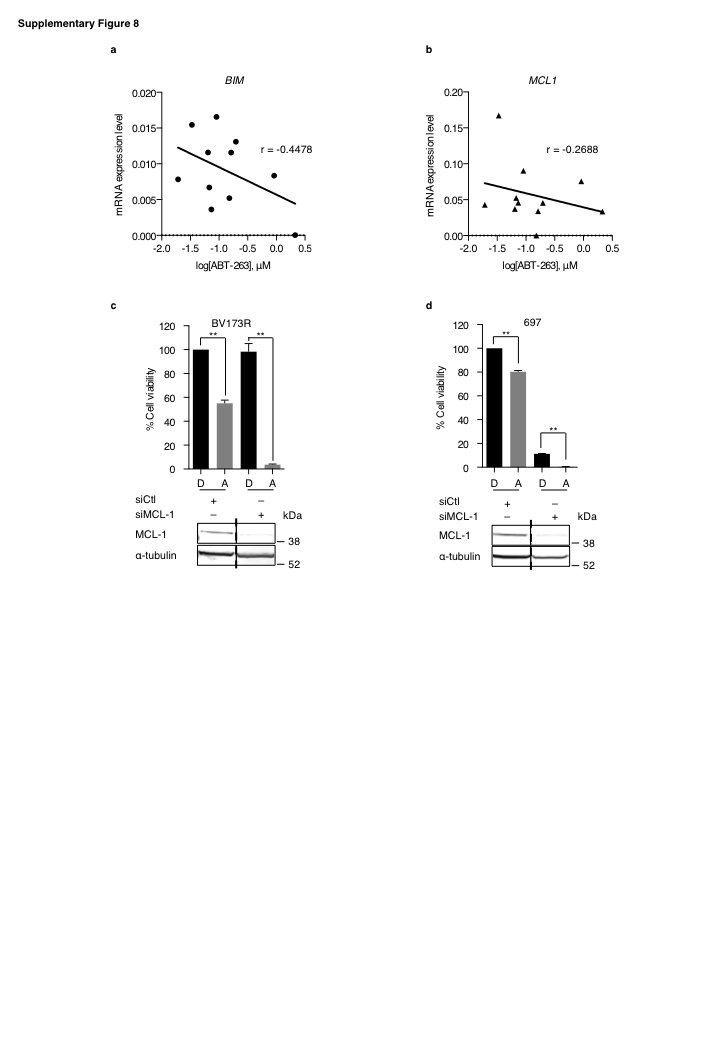


**Supplementary Figure S8. B-ALL cell sensitivity to ABT-263 is negatively correlated with BIM and MCL-1 levels. (a, b)** Graph shows Pearson correlation between *BIM* (a) or *MCL1* (b) mRNA levels and ABT-263 IC_50_s in B-ALL cell lines. Correlation coefficients are indicated by r values. **(c, d)** Graphs showing viability of BV173R (c) or 697 (d) cells 72 hours after transfection with siCtl or MCL-1 (siMCL-1) siRNAs and treatment with DMSO (D; control) or 200nM ABT-263 (A). The western blot below the graph shows MCL-1 and α-tubulin (loading control) to confirm MCL-1 knock-down. The dotted line indicates where discontinuous sections of the same blot were joined. Error bars: standard error of mean. **p<0.01.

**
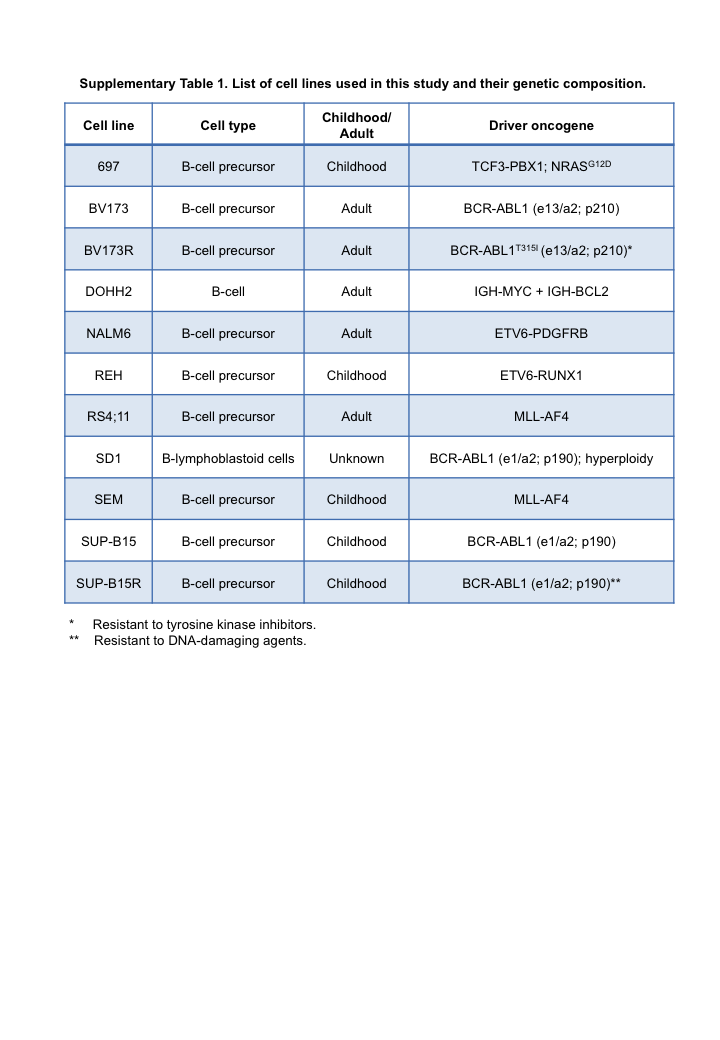
**

**Supplementary Table S1. List of cell lines used in this study and their genetic composition.** Table shows 11 B-ALL cell lines, the age category of the patient from whom the cell lines were originally derived, and their driver oncogenes.


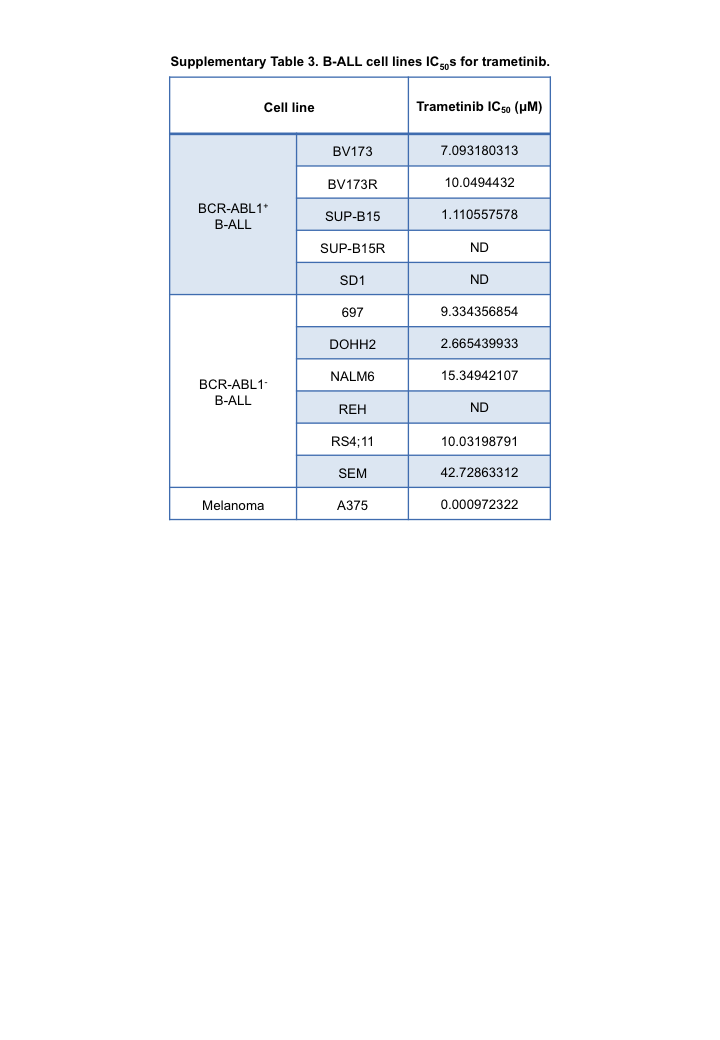


**Supplementary Table S2. B-ALL cell lines IC_50_s for trametinib.** IC_50_s are calculated from dose response curves in Figure 1d and Supplementary Figures S1c and d. A375 (BRAF^V600E^ mutant) cells are used as control. ND, not determined.

**
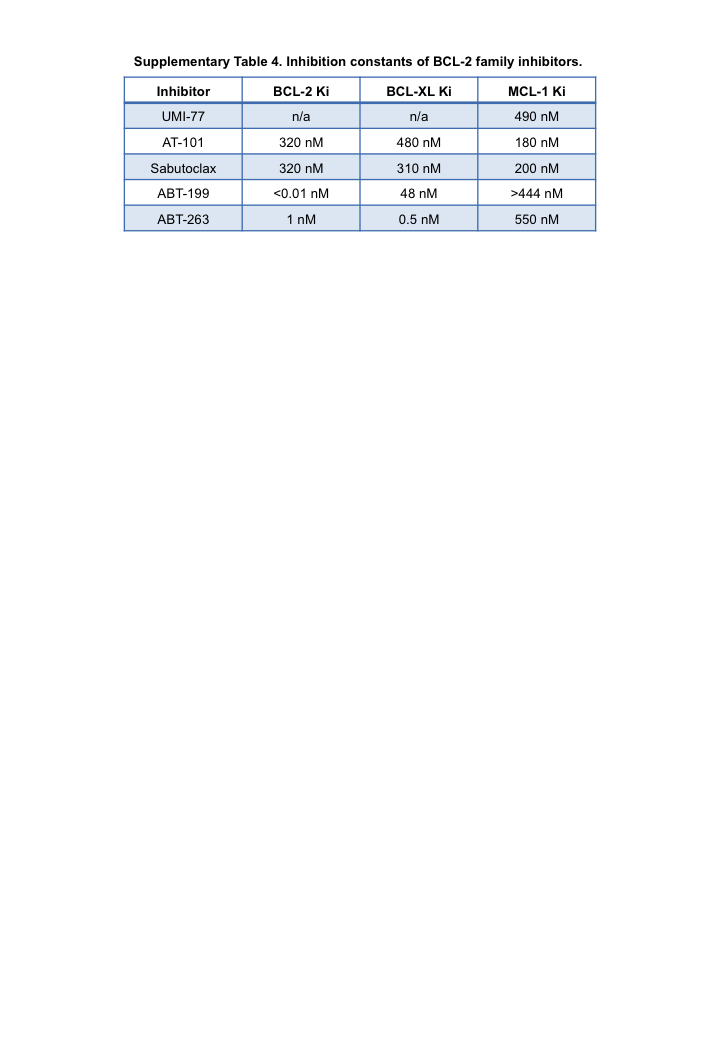
**

**Supplementary Table S3. Inhibition constants of BCL-2 family inhibitors.** Table shows inhibition constants (Ki) against anti-apoptotic proteins for UMI-77[^1^](#_ENREF_1), AT-101[^2^](#_ENREF_2), sabutoclax[^3^](#_ENREF_3), ABT-199[^4^](#_ENREF_4), and ABT-263[^5^](#_ENREF_5).


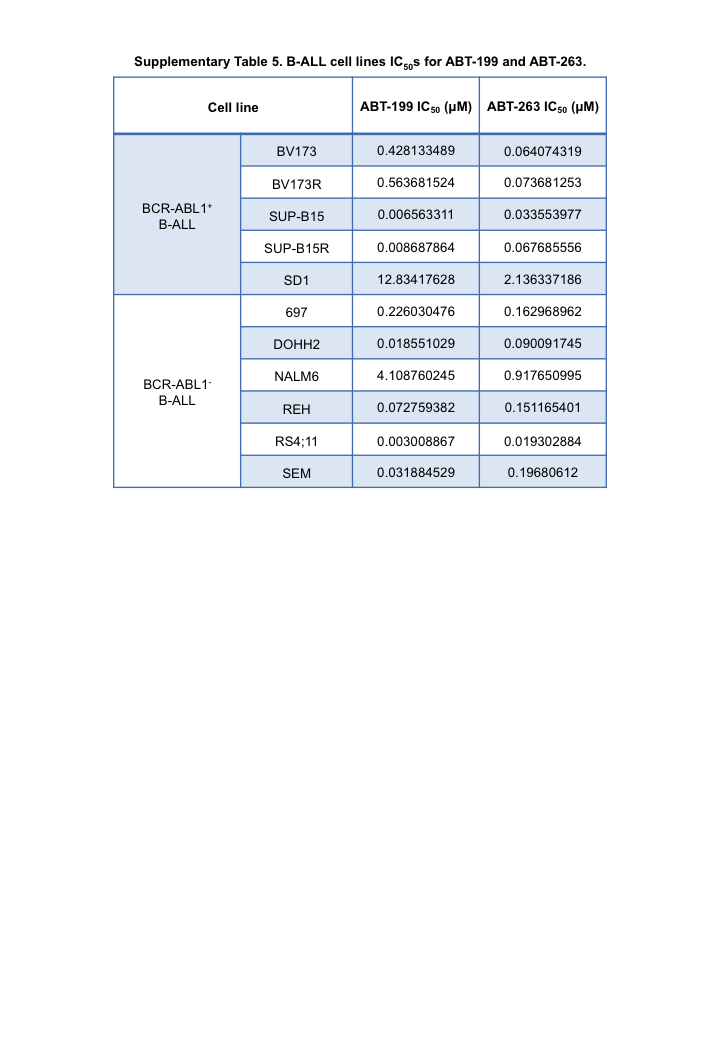


**Supplementary Table S4. B-ALL cell lines IC_50_s for ABT199 and ABT-263.** IC_50_s are calculated from dose response curves shown in Supplementary Figures S5a-b and S6a-b.

**
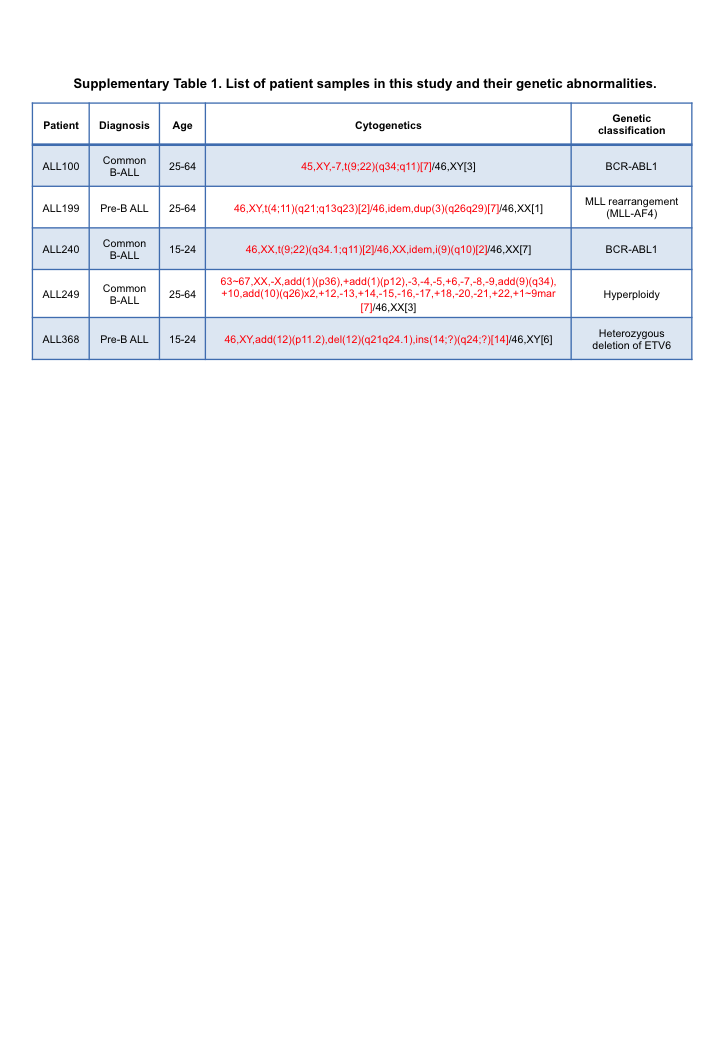
**

**Supplementary Table S5. List of patient samples used in this study and their genetic abnormalities.** Table shows the diagnosis, age group, cytogenetics analysis, and major driver oncogene or B-ALL genetic classification based on the reported cytogenetics. The abnormal cytogenetics are marked as red and the number of observed cells with the abnormalities are shown in brackets.

**References**

1. Abulwerdi F, Liao C, Liu M, Azmi AS, Aboukameel A, Mady AS*, et al.* A novel small-molecule inhibitor of mcl-1 blocks pancreatic cancer growth in vitro and in vivo. *Molecular cancer therapeutics* 2014, **13**(3)**:** 565-575.

2. Wang G, Nikolovska-Coleska Z, Yang CY, Wang R, Tang G, Guo J*, et al.* Structure-based design of potent small-molecule inhibitors of anti-apoptotic Bcl-2 proteins. *Journal of medicinal chemistry* 2006, **49**(21)**:** 6139-6142.

3. Wei J, Stebbins JL, Kitada S, Dash R, Placzek W, Rega MF*, et al.* BI-97C1, an optically pure Apogossypol derivative as pan-active inhibitor of antiapoptotic B-cell lymphoma/leukemia-2 (Bcl-2) family proteins. *Journal of medicinal chemistry* 2010, **53**(10)**:** 4166-4176.

4. Souers AJ, Leverson JD, Boghaert ER, Ackler SL, Catron ND, Chen J*, et al.* ABT-199, a potent and selective BCL-2 inhibitor, achieves antitumor activity while sparing platelets. *Nature medicine* 2013, **19**(2)**:** 202-208.

5. Tse C, Shoemaker AR, Adickes J, Anderson MG, Chen J, Jin S*, et al.* ABT-263: a potent and orally bioavailable Bcl-2 family inhibitor. *Cancer research* 2008, **68**(9)**:** 3421-3428.
